# Public Health and Hygiene

**Published:** 1896-09

**Authors:** 


### Public Health and Hygiene.

THE State Board of Health held its monthly meeting for July, at Jamestown, on Friday, July 24, 1896. There were present at the meeting: Dr. Daniel Lewis, of New York, president; Dr. B. T. Smelzer, of Albany, secretary; Hon. Theodore Hancock, attorney-general; State Engineer C. W. Adams, of Utica; Owen Cassidy, of Montour Falls, who is the legal adviser of the board; Dr. Frederick W. Smith, of Syracuse, and Frank E. Shaw, of Chautauqua county. The principal business of the session was a discussion of tuberculosis and the appointment of a tuberculosis commission, Dr. Smith and Mr. Cassidy being selected as such. This commission is charged with the inspection of cattle in this state as regards tuberculosis, the Department of Agriculture taking charge in all other contagious diseases in cattle, excepting in cases of glanders. Owing to the fact that the commission must work without adequate means, by reason of the failure of the legislature to make appropriations therefor, it will consider no cases except upon request, and then will send a veterinarian, who will decide upon the question of the presence of the disease, and if he orders the cattle killed, an appraiser appointed by the comptroller will determine the value of the property thus ordered destroyed.

Among other things, complaint was made that the hotels along the shores of Lake George emptied their sewage into the lake. This matter was considered at some length, but no definite action was taken, although it is probable that the hotels along the shores of this beautiful lake will be obliged to take care of their sewage in some other way than by carrying it to the lake.

The propriety of holding monthly meetings in various parts of the state is not to be questioned. Considerable good must certainly accrue from the familiarity with local sanitary conditions which the board will thus obtain.

---

Exact Dosage in Exercise.—Housework, gardening, chores, walking, climbing, cycling, running, swimming and many other sports, says the *Popular Science Monthly*, give just the kind of exercise that is indicated in certain conditions, due regard being had to the physiological effects of varying dosage. Oertel has shown how the simple exercise of walking may be adapted to sufferers from cardiac debility by prescribing the distance and speed and the number and lengths of the rests on definite paths, graded according to their slope. His interesting and original work has not only given a new direction to the treatment of certain cardiac affections, but is destined to have an important influence in establishing accuracy in the prescription of exercise. Whoever has studied the map of the environs of Reichenhall, Bavaria, prepared by Oertel for the application of his method, will acquire a vivid idea of what precision of dosing in exercise means. In this map the different paths suitable for the work are marked in four different colors, to indicate those that are nearly level, those slightly sloping, moderately sloping and steep, and figures are placed along each route to show the space that should be traversed in each quarter-hour. The locality itself is prepared for its remedial use by placing benches for resting at suitable distances, and by marking, on certain trees near the path, circles colored to correspond with the map, to indicate the difficulty of that particular section. By systematic practice on the easier paths heart and system are progressively trained and strengthened. Intelligent analysis may do the same work for cycling, horseback riding and many other familiar exercises. In this way the dosage is practically reduced to a definite number of kilogrammeters in a given time, and a step has been taken in placing the prescription of exercise upon a scientific basis.—*Medical Review*.

THE Indiana State Board of Health has issued a circular letter to all railroad officials asking them to have ejected from their trains every man who persists in spitting on the floor of the cars or stations after he has been warned not to do so. In the circular the board explains that the sputum contains the germs of la grippe, nasal catarrh and various other diseases. It also declares that " spitting is a nasty and unnecessary habit," and explains that the board of health will pass a rule against spitting which will have all the force of law if the railroads will post it up and endeavor to enforce it. The circular adds : " When the rule is first published and posted up in public places, this board will, of course, be loudly abused as foolish, impracticable and idiotic. Attention thus being gained, we will publish in every county reason for the action." Such a reform as the Indiana health officers have undertaken is needed in every part of the United States.—*Chicago Medical Recorder*.

